# Ternary tin-doped titanium dioxide/calcium oxide (Sn-TiO_2_/CaO) composite as a photocatalyst for efficient removal of toxic dyes†

**DOI:** 10.1039/d4ra03641g

**Published:** 2024-06-27

**Authors:** Nastaran Parsafard, Rokhsareh Abedi, Homa Moodi

**Affiliations:** a Kosar University of Bojnord, Department of Applied Chemistry North Khorasan Iran n-parsafard@kub.ac.ir +98 58 32427408 +98 58 32258865; b Electroanalytical Chemistry Research Laboratory, Department of Analytical Chemistry, Faculty of Chemistry, University of Mazandaran Babolsar Iran

## Abstract

In this study, a novel environmentally friendly route was explored for the synthesis of a tin-doped titanium dioxide/calcium oxide (Sn-TiO_2_/CaO) composite using eggshell as a ternary photocatalyst. The composite was prepared *via* a simple hydrothermal method, resulting in a unique material with potential applications in photocatalysis. The prepared photocatalysts were characterized by X-ray diffraction, Fourier transform infrared spectroscopy, UV-vis/diffuse reflectance spectroscopy, scanning electron microscopy, X-ray fluorescence, and the Brunauer–Emmett–Teller techniques. At the same time, the Sn-TiO_2_/CaO composite shows excellent degradation activity for toxic dyes. The degradation efficiencies for alizarin red, bromophenol blue, methylene blue, malachite green, and methyl red are 68.38%, 62.39%, 76.81%, 86.93%, and 17.52%, respectively, under ultraviolet light irradiation for 35 min at pH = 3. In addition, the best photocatalytic degradation efficiency for zero charge (pH 7) and basic pH is for AR 98.21% and 68.38%, MR 33.01% and 17.52%, BPB 73.17% and 17.52%, MB 72.32% and 76.81%, and MG 85.59% and 86.93%, respectively, under UV light irradiation for 35 min. The increase in photocatalytic activity of the ternary photocatalyst is accredited to the enhancement of electron–hole pair separation. Simultaneous photodegradation and photoreduction of organic dyes show that ternary photocatalysts could be used in real wastewater applications.

## Abstract 2

The present study has shown that eggshells are important as a raw material for the successful synthesis of CaO. The synthesized sample was analyzed for the photodegradation of AR, BPB, MB, MG, and MR dyes using UV light.

## Graphical abstract

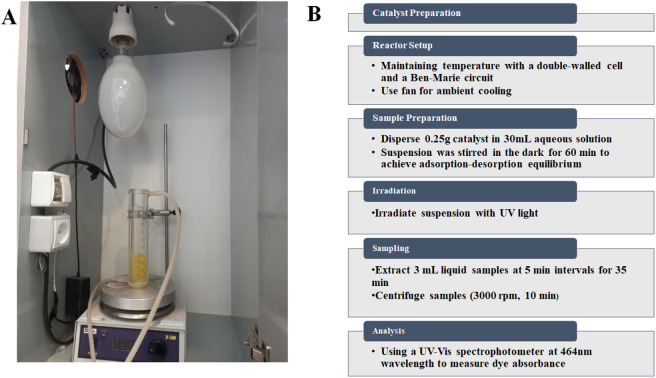

## 1. Introduction

In recent years, access to clean water has become an important issue due to rapid industrialization and urbanization. Clean water is essential for the survival of all living organisms and is therefore an important environmental and public health issue. However, the overuse of carcinogenic organic dyes in various industries such as textiles, paper, leather and pharmaceuticals has resulted in large amounts of polluting effluents entering aquatic ecosystems.^1,2^ These toxic dyes, including alizarin red (AR),^3^ bromophenol blue (BPB),^4^ methylene blue (MB),^5^ malachite green (MG),^6^ and methyl red (MR),^7^ can cause various health problems when exposed to these toxic dyes, including allergic reactions, skin irritation and respiratory problems, and also have adverse effects on the reproductive system, genotoxicity and carcinogenic properties.^8^ In addition, the release of these dyes into the environment threatens ecosystems and wildlife. Water pollution with these dyes can have long-term effects on aquatic life and disrupt the balance of natural ecosystems.^9^ Common methods to remove these dyes from wastewater streams such as flocculation, coagulation, adsorption and biodegradation are ineffective.^1^ In addition, implementing these methods often entails high costs and necessitates further programming to address the resultant by-products (lack of secondary waste formation).^12–14^ Therefore, there is an urgent need to develop a permanent, sustainable solution, and be environmentally viable for the degradation of toxic dyes in wastewater streams.

One of the most promising methods to replace the traditional and expensive methods is photocatalytic decomposition, which efficiently converts toxic dyes into non-toxic compounds, thus reducing their impact on the environment.^10^ On the other hand, the application of photocatalytic materials is a multi-step process involving the utilization of sunlight, photocatalytic properties, and the ability to recycle the photocatalyst, and it is challenging to meet all these conditions for a single material.^11^ Therefore, it is crucial to develop photocatalytic materials that integrate multiple functional components and have a constructive synergistic effect to address the above challenges simultaneously.

Among catalysts, titanium dioxide (TiO_2_) is known as the most suitable catalyst due to its low production cost, stability and non-toxicity, high photocatalytic activity, and corrosion resistance.^10,15,16^ Despite these desirable properties, its practical application is still limited due to: (1) the considerable energy band gap of TiO_2_ (3.2 eV), which limits its light absorption to the ultraviolet (UV) region; (2) the fast recombination of electron–hole pairs and the slow transfer of charge carriers; (3) difficulties in regeneration,^17–19^ The difficulties in regeneration can arise due to several factors, such as: (I) deactivation is a common issue, where the material catalytic activity decreases over time, possibly due to the accumulation of reaction byproducts or the formation of surface passivation layers,^20,21^ (II) fouling and poisoning can also occur, as impurities or unwanted products may adsorb onto the TiO_2_ surface, blocking active sites and reducing effectiveness,^22^ (III) structural changes, the TiO_2_ material can undergo structural changes, such as phase transformations or defect formation, during use,^23^ (IV) energy-intensive regeneration processes, like thermal treatment or chemical washing, may limit the practical feasibility of regeneration.^24^

Attempts have been made to solve these limitations by increasing the light absorption range and decreasing the band gap of TiO_2_.^25–28^ Several studies have focused on the synthesis of visible, photosensitive TiO_2_ by dopants that narrow the band gap and improve its photocatalytic activity.^29–32^ A promising method to achieve this is through doping with metal or non-metal ions, which serves to decrease the band gap and shift the absorption edge towards longer wavelengths (doping refers to the intentional introduction of foreign elements into the crystal structure of a semiconductor material).^33–35^ This modification allows the photocatalyst to be active under visible light, minimizing charge recombination and achieving the ideal nanoscale size for photocatalytic applications.^36^ An efficient method to increase the photocatalytic activity of TiO_2_ is doping with metal or non-metal ions. In particular, tin (Sn)-doped TiO_2_ has shown promise in reducing the band gap of TiO_2_ and increasing the photocatalytic activity.^37,38^ TiO_2_ can exist in different crystalline phases such as anatase, rutile, and brookite, each with different band gap energies, optical properties, and photocatalytic activities. Maintaining the desired crystalline phase of TiO_2_ is crucial for achieving optimal photocatalytic performance.^39^ Furthermore, reducing the particle size of TiO_2_ can significantly benefit photocatalytic processes. Smaller particles offer a greater surface-to-volume ratio, thereby increasing the available surface area for catalytic reactions. Additionally, smaller particles facilitate shorter diffusion paths for charge carriers, minimizing recombination and ultimately enhancing overall efficiency.^40^ Consequently, controlling particle size and preventing phase transformation can yield more active sites for photocatalytic reactions on the TiO_2_ surface.^41,42^

In addition, the use of calcium oxide (CaO) in photocatalysts, especially in combination with TiO_2_, offers several distinct advantages.^43^ One of the most important advantages of using CaO is its ability to increase the light absorption range of TiO_2_. Coupling (the formation of a heterogeneous structure where two different semiconductor materials are combined) between TiO_2_ and CaO leads to improved light absorption, better separation and transfer of charge carriers, and enhanced photocatalytic performance.^33,35^ In addition, the incorporation of CaO into TiO_2_ photocatalysts can improve their stability and reusability.^43^ The presence of CaO helps to prevent the accumulation of TiO_2_ nanoparticles and reduces the corrosion rate, thereby increasing the lifetime of the photocatalysts, and can also be synthesized from cheap sources such as limestone, eggshells, shell waste and calcium hydroxide.^44^

In this study, a novel environmentally friendly synthesis method was employed to create a tin-doped titanium dioxide/calcium oxide (Sn-TiO_2_/CaO) composite using eggshell as a precursor. This ternary photocatalyst, Sn-TiO_2_/CaO, was synthesized *via* a hydrothermal method. The photocatalytic activity of the catalysts prepared by photodegradation of AR, BPB, MB, MG, and MR as toxic dyes for water was evaluated in a reactor with an Hg lamp (500 W) as an ultraviolet (UV) light source right in the center. The results showed that the Sn-TiO_2_/CaO composite exhibited excellent degradation activity for toxic dyes. The degradation efficiencies for AR, BPB, MB, MG and MR are 68.38%, 62.39%, 76.81%, 86.93%, and 17.52%, respectively, under UV light irradiation for 35 min. In addition, the best photocatalytic degradation efficiency for zero charge (pH 7) and basic pH is for AR, 98.21% and 68.38%, MR 33.01% and 17.52%, BPB 73.17% and 17.52%, MB 72.32% and 76.81%, and MG 85.59% and 86.93%, respectively, under UV light irradiation for 35 min. Also, the use of eggshell as a starting material showcases the potential for utilizing waste products in the production of advanced materials with promising environmental applications.

## 2. Experimental section

### 2.1. Materials and characterizations

All chemicals were of analytical reagent grade and were used without further purification. Titanium tetrachloride (TiCl_4_), tin(iv) chloride (SnCl_4_), dichloromethane, alizarin red (AR), bromophenol blue (BPB), methylene blue (MB), malachite green (MG), and methyl red (MR) were purchased from Sigma-Aldrich. Compositional and morphological analysis were performed using Fourier transform infrared spectra (FT-IR; Nicolet iS 10 FTIR spectrometer), X-ray diffraction measurements (XRD; Philips PW1730 with CuK_α_ radiation), UV-vis/diffuse reflectance spectroscopy (UV-vis/DRS; Evolution 300 UV-vis spectrophotometer), scanning electron microscopy (SEM; VEGA3), X-ray fluorescence (XRF; XRF-8410 Rh 60 kV) and Brunauer–Emmett–Teller (BET; BELSORP Mini II). The details of these characterization methods have been mentioned and also as “ESI†”.

### 2.2. Procedure

#### 2.2.1. Preparation of Sn-TiO_2_/CaO

**Scheme 1:**
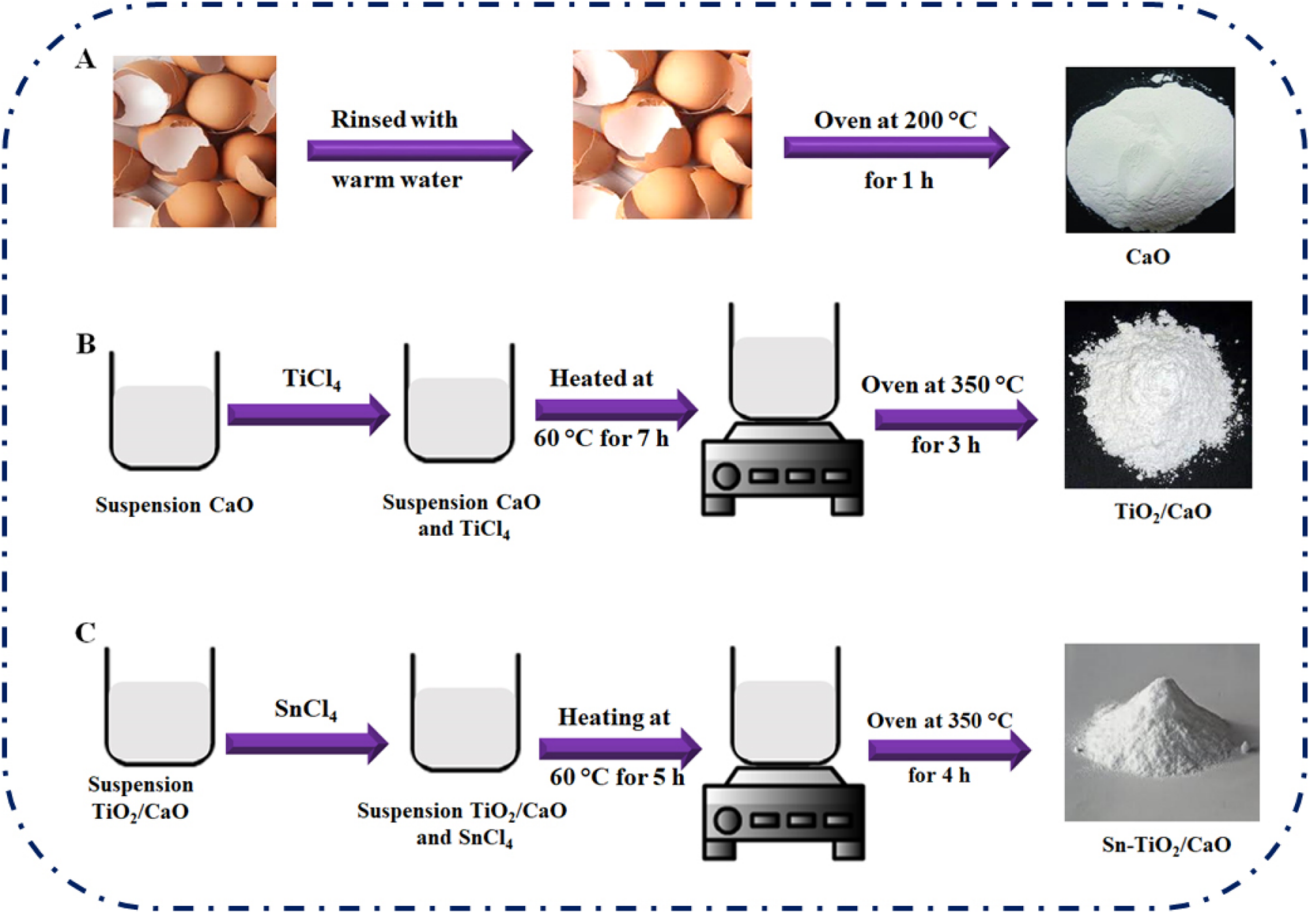
Synthesis steps of CaO (A), TiO_2_/CaO (B), and Sn-TiO_2_/CaO (C) materials.

The eggshells underwent a series of processing steps to remove impurities and create a suitable source of CaO. First, they were rinsed several times with warm water and then air-dried at room temperature for 24 h. The eggshells were then washed again with deionized water, dried in an oven at 200 °C for 1 hour and ground to produce a white solid that served as a CaO source. To facilitate the deposition of TiO_2_ on the surface of the eggshell powder, a certain amount (0.47 g) of TiCl_4_ was added to a mixture of deionized water and eggshell powder (0.50 g). This mixture was then stirred and heated at 60 °C for 7 h to obtain a dried gel, which was then dried in an oven at 350 °C for 3 h. A solution was then prepared with an optimized concentration of SnCl_4_ (0.14 g) and dichloromethane (10 mL) with a Sn/Ti molar ratio of 0.5. This solution was combined with the solid TiO_2_/CaO powder. The resulting suspension was stirred and heated at 60 °C for 5 h to obtain a gel, which was then dried at 110 °C for 8 h. Finally, the dried precipitate was calcined in an oven at 350 °C for 4 h. The catalysts prepared by these processes were designated as Sn-TiO_2_/CaO, as shown in Scheme 1.

### 2.3. Photocatalytic test

**Scheme 2:**
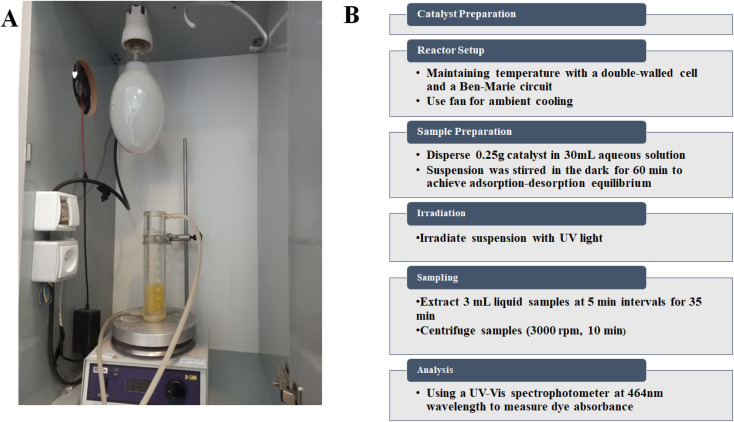
Picture of the photoreactor used (A), and flowchart the research methodology (B).

The photocatalytic activities of the prepared catalysts were investigated by the photochemical degradation of AR, BPB, MB, MG, and MR (10 ppm) as a model pollutant for water, in a reactor with a 500 W Hg lamp as an ultraviolet (UV) light source in the center of a dark box (Scheme 2A). To maintain the temperature of the solution in the presence of the lamp, a double-walled cell connected to the bain-marie circuit was used. In addition, ambient cooling was facilitated by a fan in the dark room. Since the photocatalysis process takes place on the surface of particles, the activity of the photocatalyst is strongly influenced by the pH of the solution, the type of pollutant, and the ability of the surface to adsorb pollutants. Previous reports have shown that the pH of the solution can significantly affect the photocatalytic reactions in water pollution, as it can influence the surface properties of the photocatalyst, the distribution of charge on the pollutant molecules and the formation of reactive oxygen species (ROS). Therefore, this investigation focused on two key parameters affecting the photodegradation process, namely pH (between 3 and 11) and process duration (between 0 and 35 min).

Each catalyst was dispersed in an amount of 0.25 g in 30 mL of the corresponding aqueous solution at a specific pH value. The suspension was then stirred in the dark for 60 min to achieve adsorption–desorption equilibrium. The suspension was then subjected to irradiation, with 3 mL of the liquid extracted at 5 min intervals and subsequently centrifuged (3000 rpm for 10 min) for the entire 35 min process duration. After centrifugation, the solution was filtered with filter paper to obtain a clear solution. The clear solution was then analyzed by a spectrophotometer (Evolution 300 UV-vis spectrophotometer) at the wavelengths of 591, 438, 667, 617, and 443 nm to measure the absorbance of the dyes AR, BPB, MB, MG, and MR, respectively (flowchart of method steps Scheme 2B).

### 2.4. The effective factors on photocatalytic degradation

Appropriate experiments were carried out to investigate the optimal conditions for photocatalytic degradation of AR, BPB, MB, MG, and MR dyes. The effect of pH on the photocatalytic degradation of Sn-TiO_2_/CaO in the range of 3–11 was investigated. In addition, the effect of photocatalytic degradation time of Sn-TiO_2_/CaO was examined. To investigate the influence of this multivariable system (including pH and process time), the Response Surface Methodology (RSM) with Central Composite Design (CCD) were used on the basis of 13 test runs. The response (photocatalytic degradation efficiency (PDE) of the dyes) was analyzed using Design Expert version 11 software to find the optimal conditions.

## 3. Results and discussion

### 3.1. Characterization of the different synthesized compounds

**Fig. 1:**
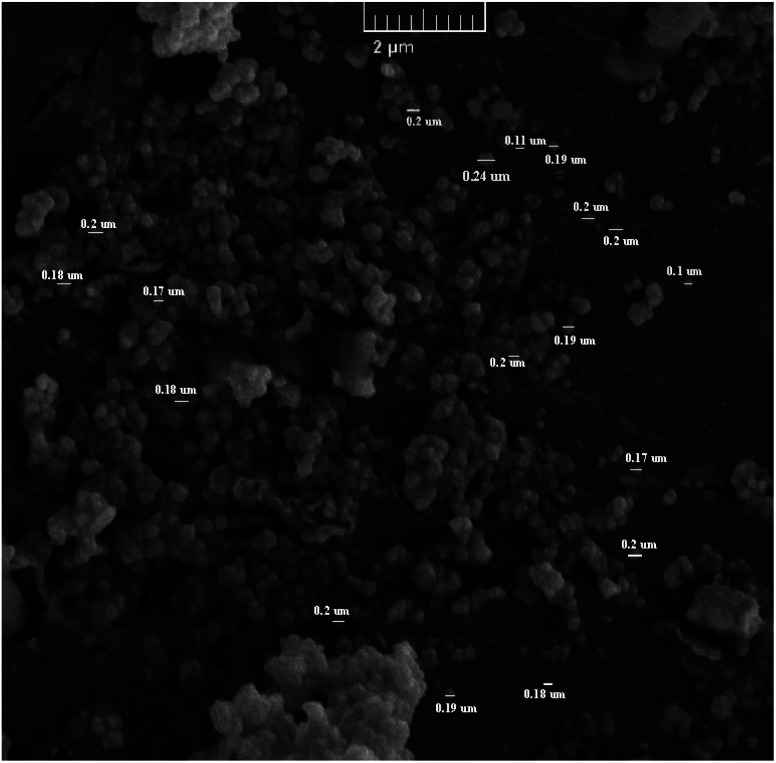
SEM image of Sn-TiO_2_/CaO.

The characterization of the different synthesized compounds is essential to understand their properties and potential applications. Scanning electron microscopy (SEM) is a very effective technique for analyzing surface morphology at the nanometer scale. In this study, the morphology of Sn-TiO_2_/CaO was investigated using SEM and shown in Fig. 1. The results show that Sn-TiO_2_/CaO has small spherical particles with an average size of 0.18 ± 0.03 nm.

**Fig. 2:**
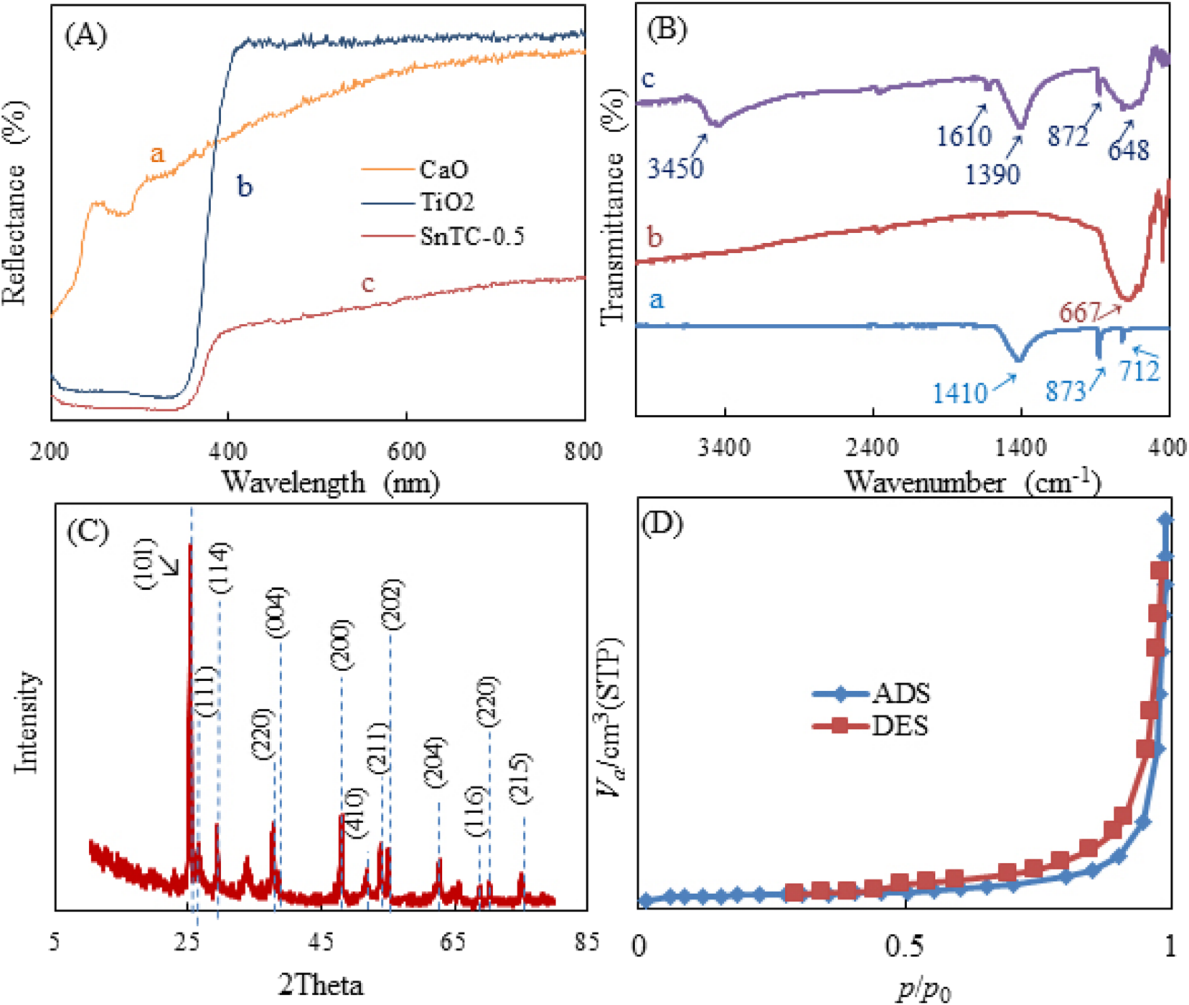
(A) UV-vis DRS of CaO (a), TiO_2_ (b), and Sn-TiO_2_/CaO (c), (B) FT-IR spectra of CaO (a), TiO_2_ (b), and Sn-TiO_2_/CaO (c), (C) XRD patterns of Sn-TiO_2_/CaO, and (D) N_2_ adsorption–desorption isotherms of Sn-TiO_2_/CaO.

UV-Vis diffuse reflectance spectroscopy (UV-Vis DRS) is a technique for quantifying the absorption of the surface and interior of a sample by the reflection of light. It is also used to analyzed the structure and composition of opaque materials. Fig. 2A, curve (a) shows the CaO spectrum. It starts with a low reflectance at about 200 nm and then rises sharply as the wavelength approaches about 220 nm. From then on, it gradually increases further and flattens out somewhat at 800 nm. Overall, CaO shows a consistently low reflectance across the entire spectrum. As can be seen in the spectrum (Fig. 2A, curve b) of this figure for TiO_2_, the reflectance starts at a low value at around 200 nm and increases sharply at a wavelength of around 350 nm, particularly in the UV range, with a fairly constant upward trend across the entire curve.^45^ The Sn-TiO_2_/CaO spectrum curve (Fig. 2A, curve c) starts with a low reflectance at 200 nm and as the wavelength approaches about 359 nm, it increases with a fairly constant trend along the graph. As seen, the Sn-TiO_2_/CaO spectrum shows a significantly lower reflectance across the visible light region compared to the TiO_2_ spectrum (Fig. 2A, curve b). This observation suggests that the doping of TiO_2_ with Sn successfully sensitized the material to visible light absorption. In general, TiO_2_ has a wide band gap, which leads to high reflection and poor absorption of visible light. However, doping TiO_2_ with Sn changes the optical properties of the material and reflects less radiation in visible light. This is a critical result, as it demonstrates the effectiveness of the doping strategy employed to improve the visible light utilization of the Sn-TiO_2_/CaO composite.

The change in material composition is obviously associated with changes in the characteristic pattern of absorption bands observed in their FT-IR spectra. Fig. 2B, curve (a) shows the CaO spectra. The bands at 1410, 873, and 712 cm^−1^ represent the Ca–OH, Ca–O, and C–O stretching vibrations, respectively, indicating the presence of CaO.^43^ Curve (b) shows the TiO_2_ spectra, where the characteristic peaks at 667 cm^−1^ correspond to the stretching vibration of Ti–O–Ti.^45^ In the FT-IR spectroscopy images of Sn-TiO_2_/CaO (curve c), the peak at 3450 cm^−1^ and a smaller band around 1610 cm^−1^ show the presence of surface-bound hydroxyl group vibrations, which can be interpreted as a result of adsorbed moisture.^45^ The vibrations corresponding to Sn–O–Sn, and Ti–O–Sn bonds are also observed in the range between 648 and 872 cm^−1^. The vibrations of the hetero-Ti–O–Sn bond are observed around 1390 cm^−1^.^36,45^

X-ray diffraction (XRD) is a technique for identifying crystalline phases in various materials and for quantitatively analyzing these phases. XRD is used in particular because of the excellent highlighting of the three-dimensional atomic structure, which has a direct impact on the properties and characteristics of the materials. The XRD pattern of Sn-TiO_2_/CaO is shown in Fig. 2C. The observed reflection peaks at ∼26.48°, 29.33°, and 54.98° were assigned to the (111), (114), and (202) facets, respectively. This crystallographic facet corresponds to the cubic structure of CaO associated with the standard spectrum (JCPDS 00-7017-0912).^43^ Additional peaks sited at 2*θ* = 25.13° (110), 33.83° (220), and 51.88° (410) correspond to the hexagonal phase of CaCO_3_ (H_2_O) (JCPDS 083-1923).^43^ These peaks are attributed to the carbonaceous nature of calcite (CaCO_3_) caused by the carbonic reaction of Ca(OH)_2_, the formation of which is due to the hydration of clinker minerals.^43^

The diffraction peaks at 25.08°, 37.78°, 47.93°, 53.88°, 62.63°, 68.83°, 70.53° and 75.18° correspond to the (101), (004), (200), (211), (204), (116), (220) and (116) planes. These peaks are associated with the anatase phase of TiO_2_ (JCPDS 21-1272).^45,46^ The intensity of the peak corresponding to the (101) plane increases along with the low intensity of the anatase peaks corresponding to the (004) and (200) planes in the Sn-doped TiO_2_ samples, indicating that Sn doping favored the crystallite along the (101) plane (JCPDS 41-1445).^46^

The Brunauer–Emmett–Teller (BET) technique is used to determine the pore size distribution and specific surface area of the samples. Nitrogen adsorption studies were also employed to characterize the N_2_ adsorption–desorption isotherms of Sn-TiO_2_/CaO and to show the BJH distributions of Sn-TiO_2_/CaO are shown in Fig. 2D. The Sn-TiO_2_/CaO exhibited a well-defined type III isotherm, typical of poor interactions between adsorbate and adsorbent materials.^47,48^Table 1 shows the surface area, pore volume, and pore size of the Sn-TiO_2_/CaO synthesized compound.

**Table:** The surface area, total pore volume, and pore sizes calculated from BET studies for Sn-TiO_2_/CaO

Catalyst	Surface area (m^2^ g^−1^)	Pore volume (cm^3^ g^−1^)	Pore size (nm)	Sn/Ti^a^
SnTC-0.5	66.577	0.033	48.289	0.491

a From XRF analysis.

### 3.2. The effect of initial pH and optimal time on photocatalytic activity

**Fig. 3:**
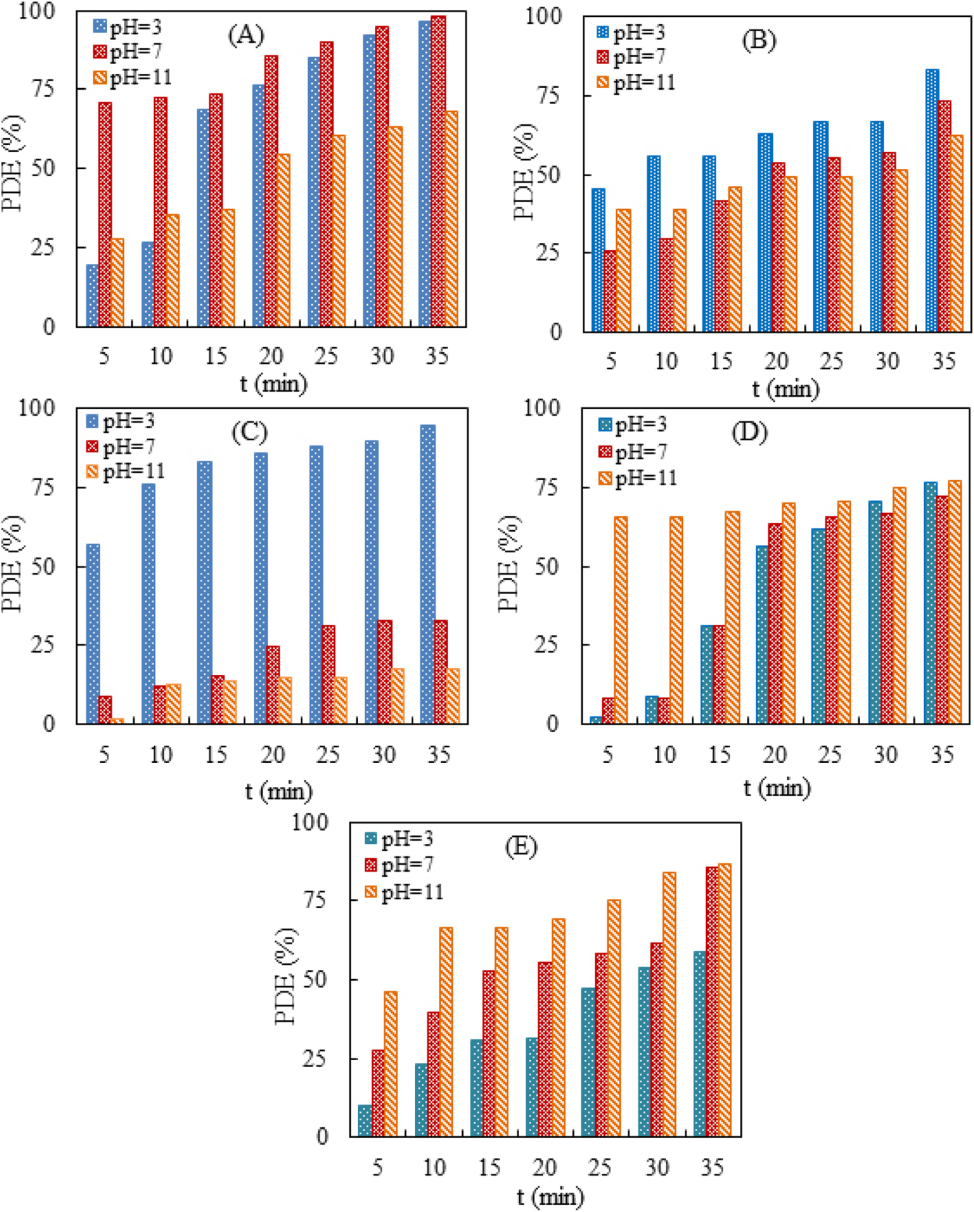
The effect of pH on the PDE% (A) AR, (B) BPB, (C) MR, (D) MB, and (E) MG in the ranged from 5–35 min.

**Scheme 3:**
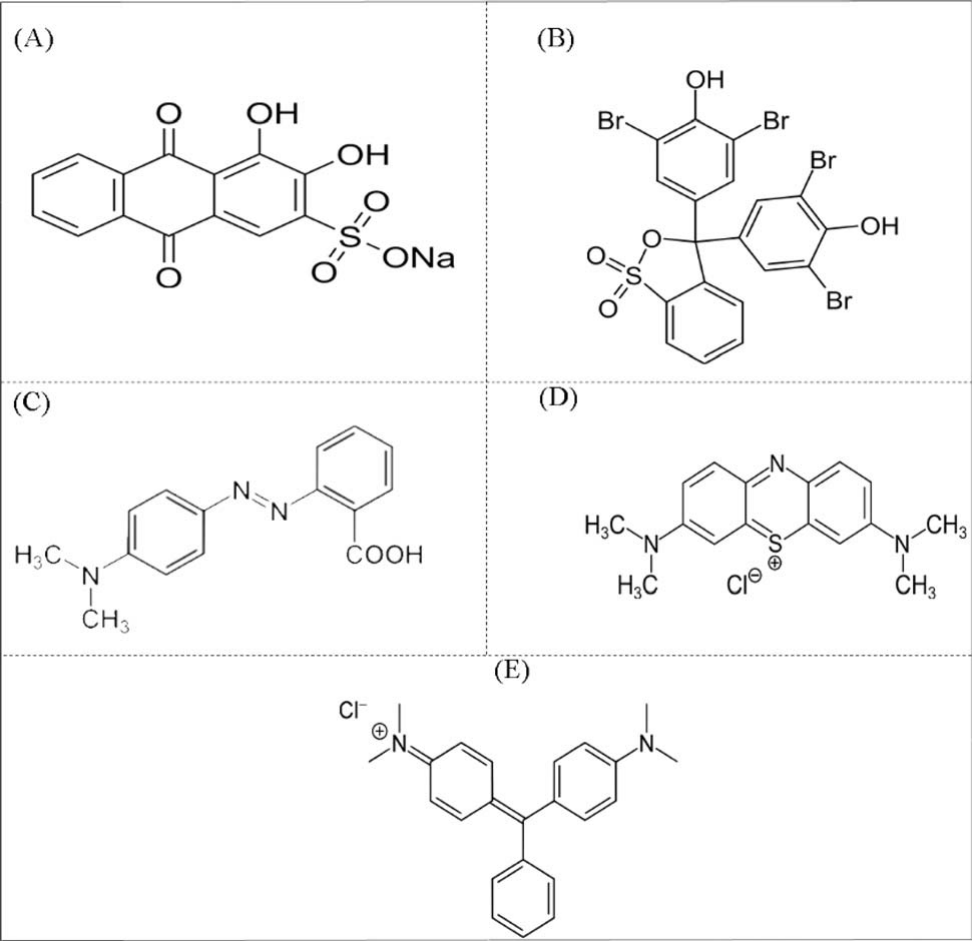
The structure of (A) AR, (B) BPB, (C) MR, (D) MB, and (E) MG dyes.

In the present study, the influence of solution pH on the photocatalytic activity of Sn-TiO_2_/CaO was investigated. The initial pH was varied in the range of 3.0–11.0, and the process duration ranged from 0 to 35 min. The pH solutions were adjusted with 1.0 M NaOH and 1.0 M HCl before the reaction and were not controlled during the reaction. The results showed that the PDE% for the solution of AR increased significantly at acidic and zero charge (pH 7), as shown in Fig. 3A. This result may be due to the protonation of the carbonyl groups (C=O) in the structure of anthraquinone AR, which increases the electrophilicity of the carbon atom (Scheme 3A). As a result, the dye structure becomes more sensitive to nucleophilic attack by active species generated during the photocatalytic process, such as hydroxyl radicals (˙OH). In addition, protonation facilitates the cleavage of aromatic rings and leads to more effective dye degradation. In a zero charge environment, the photocatalytic process can also generate ˙OH radicals that effectively attack the dye structure.

In addition, the degradation of AR was studied at different time intervals, and it was found that the optimal time of 35 min resulted in the highest PDE% for acidic and zero charge. In addition, the PDE% for the solution of BPB increased significantly at acidic pH, as shown in Fig. 3B. This result may be due to the protonation of the phenolic hydroxyl groups of the BPB molecule, which leads to the formation of positively charged cationic species (Scheme 3B). As a result, this cationic species is more susceptible to nucleophilic attack by reactive species such as ˙OH during the photocatalytic process. In addition, the BPB structure suffers from electron deficiency after protonation of the phenolic groups, which increases the ability of the reactive species to interact with and degrade the dye.

The optimum time of 35 min gave in the highest PDE% for MR at acidic pH (Fig. 3C). The results showed that the PDE% for the MR solution increased significantly at acidic pH, as shown in Scheme 3C. The MR structure consists of a benzene ring, an azo group (–N=N–), and a di-methyl-aminobenzene group (Scheme 3C). In the acidic solution, the azo group (–N=N–) of the MR molecule is protonated and forms a cationic species with a positive charge. This increases the electrophilicity of the azo group and makes it more sensitive to nucleophilic attack by reactive species such as ˙OH, which are formed during the photocatalytic process. In addition, the breaking of the MR azo bond is facilitated by its protonation, which is an important step in the degradation of azo dyes.

The optimal time of 35 min resulted in the highest PDE% for MB at basic pH (Fig. 3D). The results showed that the PDE% for MB solution increased significantly at acidic and basic pH, as shown in Fig. 3D. The MB structure consists of a central phenothiazine ring with a dimethylamino group and a sulfur atom (Scheme 3D). In an acidic solution, the central sulfur atom in the phenothiazine ring of the MB molecule can be protonated and form a positively charged cationic species. This protonation increases the electrophilicity of the sulfur atom and makes it more susceptible to nucleophilic attack by reactive species, such as ˙OH, which are formed during the photocatalytic process. Moreover, protonation facilitates the cleavage of the phenothiazine ring, which is a crucial step for the degradation of the MB dye in an acidic solution. In addition, in a basic solution of the phenothiazine ring, forming an anionic species with a negative charge is formed. Anionic species are also susceptible to attack by active species generated during the photocatalytic process, such as ˙OH. The photocatalytic process generates active species such as OH radicals in both acidic and basic media, which can effectively attack the dye structure and facilitate the degradation of MB. The optimal time of 35 min resulted in the highest PDE% for MB in acidic and basic pH (Fig. 3D). The results showed that the PDE% for the MG solution increased significantly at zero charge and basic pH, as shown in Fig. 3E. The MG structure consists of a central carbon atom bonded to three phenyl rings, one of which contains a dimethylamine group (Scheme 3E). In a zero charge solution, the central carbon atom of the MG molecule does not undergo significant protonation or deprotonation. However, the photocatalytic process generates reactive species such as ˙OH, which can interact effectively with the dye structure. In the basic solution, the central carbon atom of the MG molecule can be deprotonated and form a more reactive and negatively charged species. Deprotonation of the central carbon atom increases the sensitivity of the dye structure to nucleophilic attack by active species such as ˙OH generated during the photocatalytic process. The optimal time of 35 min resulted in the highest PDE% for MG at zero charge and basic pH (Fig. 3E). The results indicate that the photocatalytic degradation efficiency of Sn-TiO_2_/CaO is directly proportional to the pH of the dye solution and the degradation time, as shown in Fig. 3.

### 3.3. Model fitting and statistical analysis

In this study, the CCD design and the RSM approach were applied to optimize the reaction conditions. Table 2 shows the experimental matrix design under the influence of different parameters for the photodegradation of AR dye on Sn-TiO_2_/CaO catalyst. A second-order polynomial equation is shown to fit the experimental results of CCD. The following quadratic equation can express the relationship between the response and all input variables:

1 *y* = −42.1337 + 13.05091*A* + 6.64996*B* − 0.15079*AB* − 0.86048*A*^2^ − 0.08409*B*^2^

In this equation, the AR PDE (*y*) for the influence of the pH value of the sample and the time is represented in the form of coded values *A* and *B*, respectively.

CCD results by UV-irradiated Sn-TiO_2_/CaO catalysts for photodegradation of AR dye

**Table:** 

Independent variables	Units	Coded low	Coded high
pH	—	−1.0 ↔ 3.0	+1.0 ↔ 11.0
Process time (≡*t*)	min	−1.0 ↔ 0.0	+1.0 ↔ 35.0

**Table:** 

Run	pH	*t* (min)	PDE (%)
1	7.0	20.0	85.4
2	1.3	20.0	72.9
3	12.6	20.0	41.5
4	11.0	35.0	68.4
5	7.0	20.0	85.5
6	7.0	20.0	85.4
7	7.0	20.0	85.5
8	11.0	5.0	27.9
9	3.0	5.0	19.8
10	7.0	0.0	0.0
11	3.0	35.0	96.5
12	7.0	41.2	100.0
13	7.0	20.0	85.5

**Fig. 4:**
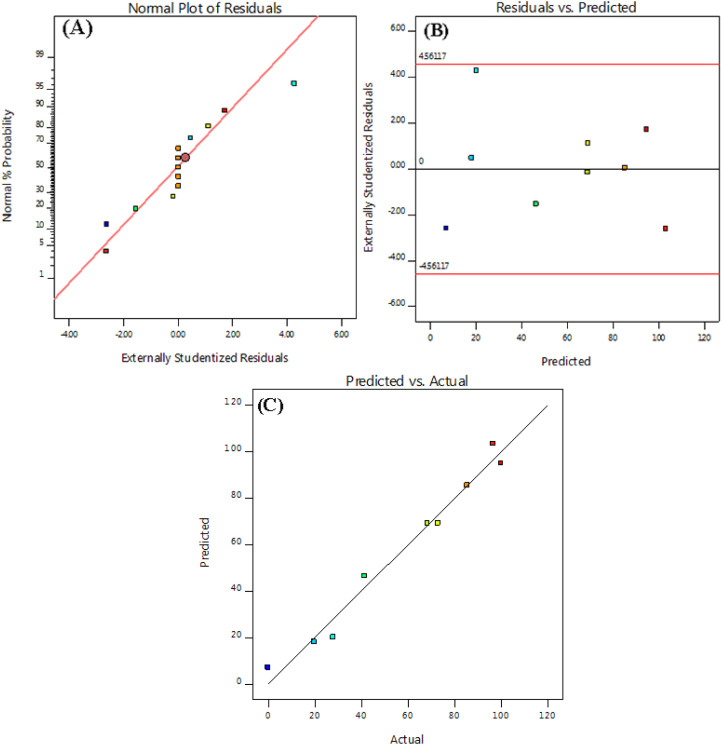
The experimental PDE values plotted against the predicted AR dye PDE values obtained from the model; (A) normal plot of residuals, (B) residuals *vs.* predicted values, and (C) predicted *vs.* actual values.

The analysis of variance (ANOVA) for the quadratic model of the predicted response surface was also calculated and is shown in Table 3. In general, Fisher's *F*-test results, *F*-values, and *p*-values are used to assess the significance of the individual coefficients and the overall model. Smaller *p*-values and larger *F*-values indicate of more significant terms within the model. The “lack of fit” means that the lack of agreement is not significant in relation to the pure error. The statistical significance and the quality of the polynomial model equation were statistically determined by the F-test within a confidence interval of 95% and the coefficient of determination *R*^2^, respectively. When evaluating the correlation between the experimental data and the polynomial regression model, a higher value of *R*^2^ indicates of a strong relationship and is therefore often considered desirable. In this particular study, the “Predicted *R*^2^” and the “Adjusted *R*^2^” were calculated to be 0.875 and 0.9695, respectively, showing a reasonable level of agreement between the two values. In addition, diagnostic plots were used to assess the appropriateness of the chosen model. The normal probability plot presented in Fig. 4 shows a uniform and almost linear distribution of the residuals. In addition, Fig. 4 shows the distribution of the residuals over the number of runs. Overall, these results indicate the necessity of the quadratic model and confirm that the experimental data were effectively captured by the model.

**Table:** ANOVA results for the quadratic model

Source	Sum of squares	df	Mean square	*F*-Value	*p*-Value
Model (significant)	12 341.96	5	2468.39	77.27	<0.0001
*A* − pH	517.96	1	517.96	16.21	0.0050
*B* − *t*	8679.35	1	8679.35	271.69	<0.0001
*AB*	327.42	1	327.42	10.25	0.0150
*A* ^2^	1323.42	1	1323.42	41.43	0.0004
*B* ^2^	2290.66	1	2290.66	71.71	<0.0001
Residual	223.62	7	31.95		—
Lack of fit	223.62	3	74.54	—	—
Pure error	0.00	4	0.00	—	—
Cor total	12 565.58	12	—	—	—
*R* ^2^	0.98	—	Std. dev.	5.65	—
Adjusted *R*^2^	0.97	—	Mean	65.73	—
Predicted *R*^2^	0.88	—	C.V.%	8.60	—
Adeq precision	25.03	—	—	—	—

In addition, diagnostic plots were used to assess the suitability of the selected model. As can be seen in Fig. 4A, the normal probability plot for the residuals is evenly distributed and almost linear. In the variance plot, the points are also randomly distributed, suggesting that the proposed model is accurate and has good precision as the residuals are between ±4.00 (Fig. 4B). Plotting the predicted and actual reaction rates for the Sn-TiO_2_/CaO catalyst shows that these points are very close to the line (Fig. 4C). All these results indicate that the quadratic model is required and the experimental data agree well with the model.

**Fig. 5:**
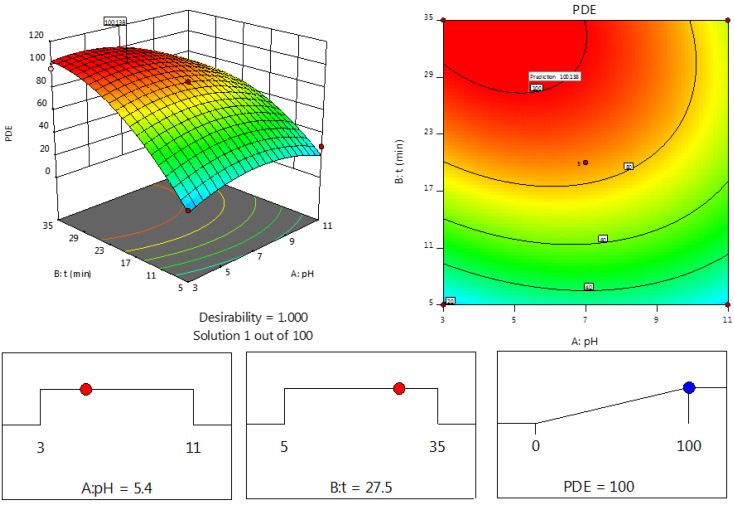
Desirability ramp for numerical optimization.

Towards the experimental design to show the effects of variables on the experimental response, the experimental design was applied to evaluate the optimal conditions. The result of the response surface is shown in Fig. 5. Since maximum PDE is the main objective for this photocatalytic degradation, the optimum conditions (100%) were determined to be pH 6.8 and a processing time of 30 min (Fig. 5).

### 3.4. Proposed mechanism of AR dye photocatalytic degradation reaction

When the Sn-TiO_2_/CaO catalyst is irradiated with light whose energy is equal to or greater than the band gap of the material, it can absorb the photons. The absorbed photons excite the electrons in the valence band of TiO_2_ to the conduction band, creating electron–hole pairs. The presence of Sn in the TiO_2_ lattice creates defect sites that can trap the photogenerated electrons and thus enhance the charge separation. The CaO component in the catalyst also provides additional adsorption sites and can facilitate the transfer of photogenerated electrons to adsorbed oxygen molecules. The photogenerated holes in the valence band can react with adsorbed water molecules or hydroxide ions to generate ˙OH. The photogenerated electrons in the conduction band can react with adsorbed oxygen molecules to generate superoxide radicals (˙O_2_^−^). A broad spectrum of active species becomes accessible for the degradation of AR molecules. The generated reactive species, such as ˙OH and ˙O_2_^−^, can effectively degrade the AR molecules adsorbed on the catalyst surface by various oxidation and reduction reactions, as indicated in the eqn (2)–(7).^49–51^

2 Sn-TiO_2_/CaO + *hv* → Sn-TiO_2_/CaO (e_CB_ + h_VB_)

3 Sn-TiO_2_/CaO (e_CB_) + O_2_ → Sn-TiO_2_/CaO + ˙O_2_^−^

4 Sn-TiO_2_/CaO (h_VB_) + OH^−^ → Sn-TiO_2_/CaO + ˙OH

5 ˙OH + AR → degradation products → CO_2_ + H_2_O

6 ˙O_2_^−^ + AR → degradation products → CO_2_ + H_2_O

7 h_VB_ + AR → degradation products → CO_2_ + H_2_O

The porosity of the Sn-TiO_2_/CaO catalyst surface plays a significant role in the effective adsorption of excited dye molecules on the surface caused by the oxidation of AR molecules through the self-sensitization process *via* the injection of electrons into the conduction band of the catalyst, as indicated in eqn (8) and (9).

8 AR + *hv* → AR*

9 AR* + Sn-TiO_2_/CaO → Sn-TiO_2_/CaO (e_CB_) + AR^+^

### 3.5. Comparison of efficiency

Table 4 provides a comprehensive comparison of the photocatalysis activity of the catalyst used in the present study with several other synthesized samples. The comparison shows that the current study has demonstrated significant progress in the effective removal of organic pollutants. This emphasizes the promising potential of the catalyst used in our study for solving environmental problems related to the removal of organic pollutants.

**Table:** Comparison of the photocatalytic performance of Sn-TiO_2_/CaO with related materials synthesized

Materials	Pollutant	Irradiation	Time (min)	Degradation activity (%)	Ref.
MgO–TiO_2_@g-C_3_N_4_	AR	Visible light	60	94	52
MH16–MH20^a^	AR	UV light	60	84	53
Sn-TiO_2_/CaO	AR	UV light	35	98.21 (pH 7)	This work
TiO_2_/g-C_3_N_4_	BPB	UV light	20	80.14	54
Cr-doped BFO^b^	BPB	UV light	120	80.02	55
Sn-TiO_2_/CaO	BPB	UV light	35	73.17 (pH 7)	This work
PVP/TEOS^c^	MR		40	96	56
Sn-TiO_2_/CaO	MR	UV light	35	33.01 (pH 7)	This work
ZnCdS/TiO_2_/Na-MXene	MB	Visible light	240	90.00	57
AgNPs/TiO_2_/Ti_3_C_2_T_*x*_	MB	Simulated solar light	120	96.00	58
Sn-TiO_2_/CaO	MB	UV light	35	76.81 (pH 7)	This work
SnO_2_/ZnO	MG	Visible light	150	98	59
ZnVFeO_4_	MG	Visible light	180	98	60
Sn-TiO_2_/CaO	MG	UV light	35	85.59 (pH 7)	This work

a Copper-doped manganese oxide nanomaterials.

b BiFeO_3_ (BFO).

c Polyvinylpyrrolidone/tetraethyl orthosilicate.

## 4. Conclusion

To explore a new environmentally friendly route, a simple synthesis method was applied to produce Sn-TiO_2_/CaO composite using eggshells. The physical and chemical properties of the synthesized composite were analyzed using XRD, SEM, BET, FT-IR, and UV-Vis DRS techniques. The present study has shown that eggshells are important as a raw material for the successful synthesis of CaO. The synthesized sample was analyzed for the photodegradation of AR, BPB, MB, MG, and MR dyes using UV light. The results show that the photocatalytic activity of Sn-TiO_2_/CaO under UV light source was significantly affected by pH. Also, the results show that the Sn-TiO_2_/CaO catalyst has the best photocatalytic degradation efficiency of AR, BPB, MB, MG, and MR with 68.38%, 62.39%, 76.81%, 86.93%, and 17.52%, respectively, under UV light irradiation for 35 min at pH = 3. In addition, the best photocatalytic degradation efficiency for zero charge (pH 7) and basic pH is for AR, 98.21% and 68.38%, MR 33.01% and 17.52%, BPB 73.17% and 17.52%, MB 72.32% and 76.81%, and MG 85.59% and 86.93%, respectively, under UV light irradiation for 35 min. The Sn-TiO_2_/CaO photocatalyst also shows excellent photocatalytic performance for the degradation and reduction of AR, BPB, MB, MG, and MR dyes, under ultraviolet light, indicating that the photocatalyst can be effective in real wastewater samples.

## Data availability

The data supporting this article have been included this article.

## Conflicts of interest

The authors declare to have no conflict of interests.

## Supplementary Material

RA-014-D4RA03641G-s001
