# Supplementary material for: Ternary tin-doped titanium dioxide/calcium oxide (Sn-TiO2/CaO) composite as a photocatalyst for efficient removal of toxic dyes
Source: RSC Adv. 2024 Jun 27;14(28):19984–95. doi: 10.1039/d4ra03641g (PMC11210368; doi:10.1039/d4ra03641g)
Supplement: RA-014-D4RA03641G-s001 [file RA-014-D4RA03641G-s001.pdf]

Electronic Supplementary Material (ESI) for RSC Advances.
This journal is © The Royal Society of Chemistry 2024

Electronic Supplementary Material:

1. Characterization methods

X-ray diffraction analysis (XRD) had recorded with a Philips PW1730 diffractometer with Ni filter and graphite monochromator. This device uses Cu kα radiation (wave-length = 1.15 Å) as an X-ray source. The scan area is 2θ = 10º–80º at 45 kV and 50 mA with a 0.06° 2θ-step and 1 s per step.

Fourier transform infrared (FT-IR) spectroscopy had used for the identification of chemical bonds in the prepared catalysts. FT-IR spectra had recorded on a Nicolet iS 10 FTIR spectrometer, in the wavenumber of 400–4000 cm^{-1}.

UV–Vis diffuse reflectance (UV–Vis DRS) had carried out on an Evolution 300 UV-vis spectrophotometer using $BaSO_4$ as a reference. The power catalysts had evaluated in 200–800 nm at room temperature.

To characterize the surface areas and porosity, N_2 adsorption–desorption isotherm had obtained by BELSORP Mini II using the outgassed sample under vacuum at 623 K for 10 h before the measurements. The specific surface area (SBET) and the volume of the adsorbed monolayer (V_p) had evaluated by the BET equation and by assuming an N_2 molecule to cover 0.162 nm^2, respectively. The Barret-Joyner-Halenda (BJH) method had also used for calculating the average pore diameter (d_p).

The morphology of catalyst had investigated on a VEGA3 with an accelerating voltage of 30 kV.
